# Links between self-monitoring data collected through smartphones and smartwatches and the individual disease trajectories of adult patients with depressive disorders: Study protocol of a one-year observational trial

**DOI:** 10.1016/j.conctc.2025.101492

**Published:** 2025-05-10

**Authors:** Hanna Reich, Simon Schreynemackers, Rebeka Amin, Sascha Ludwig, Jil Zippelius, Johannes Leimhofer, Tobias Dunker, Elisabeth Schriewer, Angela Carell, Yvonne Weber, Ulrich Hegerl, the MONDY consortium

**Affiliations:** a Research Centre of the German Foundation for Depression and Suicide Prevention, Department of Psychiatry, Psychosomatic Medicine and Psychotherapy, University Hospital, Goethe University, Heinrich-Hoffmann-Straße 10, 60528, Frankfurt am Main, Germany; b German Foundation for Depression and Suicide Prevention, Goerdelerring 9, 04109, Leipzig, Germany; c Institute for Applied Informatics, University Leipzig, Goerdelerring 9, 04109, Leipzig, Germany; d Institute of Computer Science, Heinrich-Heine-University, Graf-Adolf-Straße 63, 40210, Düsseldorf, Germany; e adesso SE, Business Line Health, Adessoplatz 1, 44269, Dortmund, Germany; f Department of Epileptology and Neurology, University RWTH Aachen, Pauwelsstraße 30, 52074, Aachen, Germany; g Goethe Research Professorship, Department for Psychiatry, Psychosomatics and Psychotherapy, University Hospital, Goethe University, Heinrich-Hoffmann-Straße 10, 60528, Frankfurt am Main, Germany

**Keywords:** Depression, Digital phenotyping, Passive sensing, Ambulatory assessment, Smartphone, Smartwatch, VAR-models, Machine learning, Forecasting, Proof-of-concept, n-of-1 design

## Abstract

Depression is highly recurrent and heterogenous in its individual course, requiring a personalized treatment approach. Patients today can collect large volumes of personal data via smartphones and smartwatches and may utilize them for their treatment and self-management. We aim to provide proof-of-concept that these data can (i) serve as an objective marker of and (ii) predict the daily and weekly self-reported depression severity within individuals with depressive disorders.

In this exploratory study, 15 adult patients with depressive disorders will collect self-report and biosensor data over the course of one year. Participants will (a) attend three in-person appointments (at baseline, 6 months, and 12 months), (b) self-report daily and weekly depressive symptoms, (c) continuously collect sensor data via the “iTrackDepression” app on their Android smartphone (app usage, phone calls, phonetic parameters from voice recordings), and (d) wear a Samsung Galaxy Watch 5® to record data from the accelerometer, step sensor, light sensor, and heart rate sensor. We will apply multilevel correlations, vector-autoregressive models, and Machine Learning approaches to identify individual patterns in the data, particularly in the relationships between biosensor data and self-reported depressive symptoms.

Enhancing the understanding of individual disease trajectories through data from smartphones and smartwatches could allow for classical, digital, and self-management interventions for depression to be delivered in a manner and at a time specifically tailored to the individual's needs.

Clinical trial registration number: DRKS00032618 (https://drks.de/search/en/trial/DRKS00032618)

## Highlights

• The phenotype of depressive disorders is highly heterogeneous.
• Patients are owners of large personal datasets from smartphones and smartwatches.
• Data-driven methods can generate personalized disease markers and predictive models.
• Patients with depression may use these for disease monitoring and self-management.

Depressive disorders are highly recurrent: At least 50 % of individuals who recover from a first episode of depression will have one or more additional episodes in their lifetime, and the number increases to a staggering 80 % for those with a history of two episodes [1]. Most patients with depressive disorders have an unfavorable prognosis and the majority experiences a disabling and chronic disorder [2], characterized by premature mortality [3], reduced quality of life [4], and loss of occupational functioning [5]. After acute depressive episodes have been treated, substantial, unresolved morbidity in patients with depressive disorders remains. Patients with a recurrent depressive disorder face another year to reach functional recovery after syndromal remission, which is due to residual depressive symptoms [6]. The highly recurring nature of depression and its devastating consequences for patients, their families, and the society makes the monitoring and prolonged disease management of depression an increasingly recognized priority [7]. Patients urgently require markers for monitoring their disease state and predictions about their expected disease course [8], but presently available clinical prediction models for relapse and recurrence of depression are not yet sufficiently developed for being deployed [9] and so far no clinically relevant tools for stratifying subgroups in individuals affected by depression have been established [10].

This lack of success in establishing predictive models for depression can be attributed to the highly heterogenous phenotype of depressive disorders [11]. The heterogeneity of phenotypes leads to not very informative conclusions for the individual patient, as research traditionally applied group-level statistics that are combining data from individuals with very different problems into one category [12]. Consequently, the individual patient needs to be the focus of analysis to create meaningful conclusions from their personal data [13]. The collection of rich, longitudinal datasets through ambulatory assessment (AA, using smartphones and smartwatches for data collection [14]) provides the opportunity to run statistical analyses for individual participants. In a previous study, we were able to use daily, self-reported data to reveal the specific temporal order of illness processes in single patients [15] and to create unique, personalized dynamic network models depicting the clinically well-known heterogeneity of depression [16]. These approaches may aid the identification of markers and predictors of individual disease trajectories. AA is considered less intrusive than diagnostic assessments in a laboratory or clinical environment [17] and less prone to recall bias compared to retrospective assessments [18]. However, daily self-reporting can become burdensome to participants.

Therefore, great hope for identifying markers and predictors of depressive disease trajectories has been placed in the rise of mobile sensing (MS), using build in sensors of smartphones and wearables to collect data in real time [19]. MS facilitates the collection of personal data reflecting individual behaviour and physiology that is related to depression, such as sleep [20], physical activity [21], heart rate [22], speech features [23], and smartphone use [24]. Numerous studies have shown associations between objective behavioral and physiological features collected via MS and depression [25]. However, few studies covered longer time spans that allow for the modelling of depressive illness processes within individuals over the life span. In a recent systematic review [26], we found only nine studies that had collected MS data from patients with depressive disorders for time-spans of 12 weeks or more. One study showed that sleep features (sleep architecture, sleep stability, sleep quality, insomnia, and hypersomnia) collected using a Fitbit device were associated with a worsening of the functional depressive status over the following two weeks [27]. Six out of nine studies included in our systematic review demonstrated predicting capabilities of data on activity (step count, heart rate), sleep (including circadian rhythm), communication (call logs, messaging), phone usage (screentime), and location (Global Positioning System, GPS) for depressive episodes and/or symptom severity [[28], [29], [30], [31], [32], [33]]. Based on MS data, a predictive accuracy for depression from 79 % to 91 % has been achieved [26]. One study in particular showed that in adolescents, personalized models to predict depression severity from MS data achieved better results than universal models [32]. To our knowledge, this result has not yet been replicated for adult patients with depressive disorders.

In summary, patients with depressive disorders need objective markers to monitor their illness severity and reliable, valid models to predict clinically meaningful changes in the severity of their condition. This is essential as symptom presentations can vary greatly within individuals over time [34], and those with depressive disorders experience fluctuations in both positive and negative affect [35]. Objective markers could support patients in the self-management of their illness and predictive models could inform health professionals concerning treatment decisions. As phenotypes of depression vary greatly, these markers and predictive models need to be made-to-measure for each patient. Using MS, patients can unobtrusively collect enough personal data to facilitate the creation of personalized models for data analysis. To our knowledge, however, no study has shown that personalized models based on MS data can serve as objective markers of individual illness severity or forecast depression severity for individual patients. Therefore, the current study aims to achieve a proof-of-concept showing that personal MS data can (I.) monitor and (II.) predict depression severity in individual patients. To this end, adult participants with depressive disorders will collect continuous, daily data on sleep/deep rest, physical activity (locomotion, heart rate), speech features, smartphone usage (including digital communication behaviour) as well as self-reported depressive symptom severity over the course of one year using AA methods. With this data, we aim to answer the following exploratory research questions (RQ):

I. a) Are personal behaviour (sleep/deep rest, physical activity, smartphone usage) and physiology (heart rate, heart rate variability, speech) during daytime (6 a.m.–6 p.m.) associated with self-reported depressed mood and anhedonia in the evening within adult patients with depressive disorders?
b) Are personal behaviour (sleep/deep rest, physical activity, smartphone usage) and physiology (heart rate, heart rate variability, speech) during daytime (6 a.m.–6 p.m.) of the last 7 days associated with the weekly self-reported depression severity within adult patients with depressive disorders?
II. a) Can individualized statistical models based on longitudinal data on personal behaviour (sleep/deep rest, physical activity, smartphone usage) and physiology (heart rate, heart rate variability, speech) forecast the self-reported depressed mood and anhedonia in the evening within adult patients with depressive disorders?
b) Can individualized statistical models based on longitudinal data on personal behaviour (sleep/deep rest, physical activity, smartphone usage) and physiology (heart rate, heart rate variability, speech) predict the weekly self-reported depression severity within adult patients with depressive disorders?

## 1 Methods

### 1.1 Design

This exploratory study uses an observational n-of-1 design [36], involving repeated measurements of self-reported, behavioural, and physiological outcomes over the time of one year (365 days) within individuals without manipulation of variables by the investigators. The work is carried out in accordance with the World Medical Association Declaration of Helsinki [37]. Ethical approval has been granted by the Ethics Committee of the Department of Medicine at Goethe University Frankfurt, Germany (date: 24/08/2023, reference number: 2023–1309). The study protocol has been registered in the German Clinical Trials Register (reference number: DRKS00032618).

### 1.2 Study population

We aim to collect 10–12 complete case studies of adult patients with depressive disorders; allowing for some dropout, *N* = 15 participants will be included. Patients are recruited at the Department of Psychiatry, Psychosomatic Medicine and Psychotherapy, University Hospital of Goethe University Frankfurt, Germany. This study protocol is presented to treating physicians and psychologists at various occasions and a flyer with the inclusion and exclusion criteria (see Table 1) is provided. Physicians and psychologists invite patients who are receiving treatment for affective disorders to participate in the current study. Interested patients provide their contact details and written consent to be contacted by the study centre. If sufficient participants can't be reached, various communication channels of the German Foundation for Depression and Suicide Prevention (e.g., newsletter, website, online discussion forum) shall be used to draw the attention of interested participants to the study.

**Table 1:** Inclusion and exclusion criteria for study participation.

Inclusion criteria	Exclusion criteria
- Minimum 18 years old	- Acute risk of self-hazard/suicidality (six months before inclusion)
- Diagnosis of a recurrent major depression (ICD-10 F33) with two or more depressive episodes during the last ten years	- Currently severe depressive episode with psychotic symptoms (F33.3)
- Currently residual symptoms of depression (BDI-2 sum score ≥14)	- Lifetime diagnosis of any psychotic disorder (any diagnosis according to ICD-10 F2)
- Being in concurrent treatment for the depressive illness	- Lifetime diagnosis of any bipolar disorder (ICD-10 F31)
- Being able to provide informed consent	- Lifetime diagnosis of any borderline personality disorder
- Usage of an own Android smartphone (or agreement to switch to an Android smartphone provided by the study centre)	- Severe somatic illnesses requiring intensive treatment
- In case of any lifetime substance abuse disorder: being in stable remission (min. 6 months)	

Note: ICD-10 = International Classification of Diseases, Tenth Revision, BDI-2 = Beck Depression Inventory-II.

### 1.3 Procedures

**Fig. 1:**
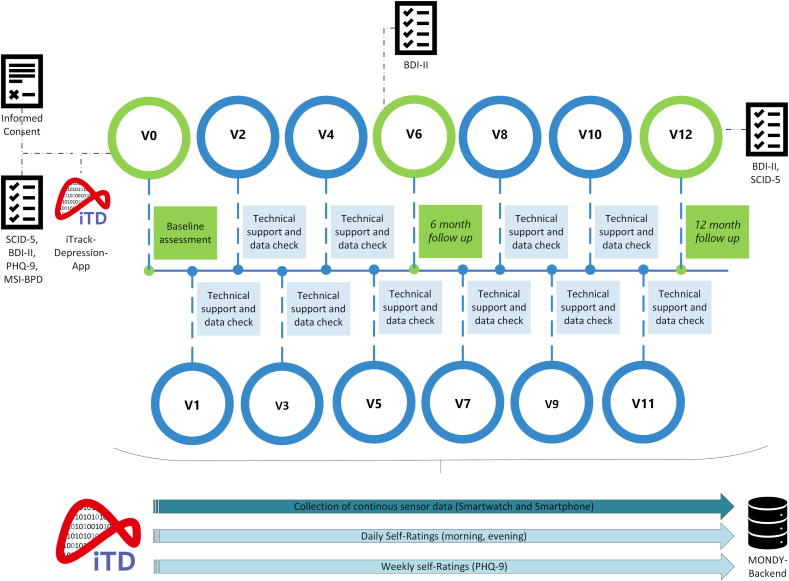
Timeline of study procedures. Caption: At baseline (V0, time requirement: 3.5–4 h), participants read the participant information sheet, clarify any questions about the study with the study team, and provide written, informed consent for study participation. A standardized clinical interview (SCID-5) is conducted and several self-report questionnaires are filled in (see Measures section). After the assessments are completed, the participants are assisted in installing the iTrackDepression-App (iTD-App) via the Google PlayStore on their smartphone. The iTD-App has been developed by the German Foundation for Depression and Suicide Prevention in collaboration with adesso SE (Dortmund, Germany) and the InfAI Institute for Applied Informatics (Leipzig, Germany). It has two major components for the collection of depression-related data: self-report assessments and sensor technology. Additionally, participants are asked to continuously wear a Samsung Galaxy 5® smartwatch that is provided by the study centre to record data on physiological and behavioural parameters such as heartbeat and physical activity (please refer to Measures for a detailed description). Any technological questions are clarified before the participants start continuous data collection in their daily lifes. V1-V5 and V7-V11 are used to provide technical support, check for data completeness, and manage suicidality if necessary. These appointments can be conducted via video call or telephone, depending on the preference of the participants. At V6 (6-month follow-up), the BDI-II will be completed at the study centre and any changes in the treatment plan during the last six months are assessed. The final appointment (V12) is conducted after 12 months of study participation. At V12, the SCID-5 is repeated to assess for any psychiatric diagnosis during the year of study participation and the BDI-II will be completed to check and record the participants' status at the end of the study. Changes in the treatment plan during the study participation are assessed and recorded. The Samsung Galaxy 5® smartwatch is returned to the study team and any final questions are answered. Note: MONDY is the name of the overarching project: “MONDY: Secure and open platform for AI-based healthcare apps”, see Funding information.

Eligible participants are screened for inclusion and exclusion criteria and then start with study participation. The participation in the study encompasses three major appointments at the study centre: Baseline (V0), 6-month follow-up (V6), and 12-month follow-up (V12). In between, shorter monthly appointments (V1-V5, V7-V11) are planned to assist in case of any technical difficulties, to assess data completeness with the participants, and to manage suicidality if necessary. Fig. 1 provides details of the timeline and study procedures.

Data completeness is monitored continuously by the study centre, supported by a custom application that provides metadata on daily questionnaire completion and duration of smartwatch wearing. Participants are contacted by the study centre if they have made less than one entry in the iTD-App or have worn the smartwatch for less than 10 h per day for three consecutive days. If the issue can't be resolved via phone, e-mail, or videocall, participants are invited for an in-person meeting at the study centre to resolve any issues. Participants receive a compensation of 200 Euro for their participation in the study (€15 per completed month, plus an additional €20 upon completion of V12 and return of the smartwatch).

#### 1.3.1 Management of suicidality

Due to the phasic nature of depressive disorders, worsening of symptoms may occur during study participation, including the occurrence of suicidal thoughts. Participants are required to be in treatment for their depression (see Table 1) as there will be no treatment or continuous, face-to-face monitoring of suicidality through the study centre. However, the weekly self-report of depressive symptoms (see Measures) includes one item assessing suicidal thoughts. During the monthly appointments, the participants’ answers to this item are reviewed. In case that a participant has indicated the presence of suicidal thoughts on more than half of the days or almost every day during the last week (item 9 ≥ 2) on one or more occasions, the project leader is informed and participants are encouraged to either contact their attending physician or psychotherapist, the nearest outpatient clinic of a psychiatric clinic, or the psychiatric emergency department. If the study participation cannot be continued safely, termination of the participation is discussed with the participant.

#### 1.3.2 Premature study termination (dropout)

Participation in the study is voluntary. Participants can decide to opt-out of the study at any time without providing any reasons. In case that the participation of a participant ends prematurely, all procedures of V12 are brought forward to a final assessment. If a participant is not able to complete V12, only a short meeting is scheduled to return the smartwatch and complete the BDI-II questionnaire.

#### 1.3.3 Trial status

Recruitment started in August 2023, first patient in was September 27, 2023, last patient out was December 02, 2024.

### 1.4 Measures

Three categories of data (clinical assessments and two types of AA data: self-report and MS) are collected and described below.

#### 1.4.1 Clinical assessments

##### 1.4.1.1 The structured clinical interview for DSM-5® (SCID-5)

The SCID-5 [38] is a semi-structured interview guide for making the major diagnoses in line with DSM-5 (Diagnostic and Statistical Manual of Mental Disorders, Fifth Edition [39]). It is administered by a trained mental health professional to assess inclusion and exclusion criteria for study participation at V0, and to assess any psychiatric diagnosis during the year of study participation at V12.

##### 1.4.1.2 Beck Depression Inventory® (BDI-II)

The BDI-II [40] is a brief, self-report inventory designed to measure the severity of depression symptomatology. It is administered by a trained mental health professional at V0, V6, and V12. To assess the presence of residual depressive symptoms, a sum score of ≥14 is used as inclusion criterion.

##### 1.4.1.3 McLean screening instrument for borderline personality disorder (MSI-BPD)

The MSI-BPD [41] was developed as a self-report screening measure for DSM-IV (Diagnostic and Statistical Manual of Mental Disorders, Fourth Edition) borderline personality disorder [42]. It is administered by a trained mental health professional at V0 with a cut-off score of ≥7 to exclude individuals with potential borderline syndrome.

#### 1.4.2 Participant self-report

Daily (morning, evening) and weekly self-reports are assessed through the iTD-App. The exact wordings of all daily self-report items are shown in the Supplementary Material.

##### 1.4.2.1 Daily self-report

In the morning and evening assessments, well-being (mood, tension, exhaustion, rumination) and sleep in the previous night are assessed. In the evenings, daily self-reported depressive symptom severity is assessed using the Patient Health Questionnaire-2 (PHQ-2 [43]), a very short measure assessing two core symptoms of depression (loss of interest/joylessness and feeling down/depressed/hopeless). Following previously established procedures [15,16], answers are given on a Visual Analogue Scale ranging from 0 to 10 with labelled endpoints (0 = never, 10 = all the time). Additionally, contextual information is queried to facilitate the interpretation of the data (substance use, medication, daytime activities).

##### 1.4.2.2 Weekly self-report

The Patient Health Questionnaire-9 (PHQ-9, [44]) is used to assess the weekly self-reported depressive symptom severity. To this end, the original wording of the introductory sentence was changed to “Over the *last week*, how often have you been bothered by any of the following problems?“. The PHQ-9 is a short self-report measure of depressive symptoms that is able to detect changes in depression over time [45] and has been recommended as a general measure of depression severity by the DSM-5 [39]. Each item is rated on a four-point Likert scale ranging from 0 to 3, total scores range from 0 to 27. Higher scores indicate more severe depressive symptoms.

The Situational Self-Awareness Scale (SSAS, [46]) is used to assess weekly fluctuations in public and private self-awareness occurring at the time of self-monitoring during this study. Data can be used to clarify the relation between self-awareness, continuous self-monitoring, and depressive symptoms. The SSAS is sensitive to changes in self-awareness within individuals over time and across situations [46]. A German version is used which was created by two native German speakers using the double-translation and reconciliation procedure [47].

Additionally, participants are asked weekly about life events (“Were there any special events this week that concern you?“), with the option to enter a free text note in the iTD-App.

#### 1.4.3 Mobile sensing

Objective parameters are generated by MS technology, connecting external biosensors to the iTD-App and by evaluating smartphone-internal sensors and the operating system.

##### 1.4.3.1 Biosensors

Participants collect continuous data with portable sensors. A SAMSUNG Galaxy Watch5® is connected to the participant's smartphone via Bluetooth. The smartwatch includes advanced and reliable sensor combinations; the 3-axis accelerometer, motion sensor for step count, light sensor, and optical heart rate sensor are used and recorded by the iTD-App. Data from the Samsung smartwatch is obtained via the Samsung Privileged Health SDK (Tracking Service) and WearOS sensors, temporarily stored on the watch's local database, then iteratively transferred to the smartphone's local database. The data is subsequently uploaded to the MONDY platform, with no involvement of external providers.

##### 1.4.3.2 Internal sensors of the smartphone

The smartphone's microphone is used for daily voice recordings. Participants are provided with daily varying stimulus material from a set of 16 questions (see Supplementary Material) to make voice recordings of 30 s within the iTD-App. For this purpose, up to four questions are offered as stimulus material. The questions are randomly selected daily to provide variety. Voice recordings are processed to extract phonetic parameters (e.g. frequency, pitch), but will NOT be analysed in terms of content (i.e., no natural language processing, NLP).

##### 1.4.3.3 Data from the operating system

Data on the active use of the smartphone is routinely collected from the operating system of the smartphone itself. This data is collected through the iTD-App, limited to: app usage (e.g., social media, gaming apps, other apps; recorded in usage time and data volume), messaging behavior (time and data volume spend on messaging), and phone calls (duration and amount of incoming, outgoing and missed calls). In addition, called contacts are recorded anonymously. For this, the iTD-App assigns a hash code to each phone number and collects the information about which hash code has been contacted through calls. No personal data about the contacts (such as the phone numbers and names of the contacts) is recorded.

### 1.5 Data management and privacy

The legal basis of the data processing is the voluntary consent (Art. 6 (1) (a) GDPR): Data is only collected and included if participants consent to data processing by written informed consent after having read the participant information and data processing information. Permissions are also requested within the iTD-App for the use of the individual sensors.

Upon first contact with the study centre, a Participant Identification Code (PIC) is assigned to each interested participant. All data collected during the study is stored pseudonymized using this PIC. A list with all study participants, i.e. their name, PIC and status (included/excluded, study ongoing/discontinued/completed) is kept confidentially at the study centre. The PIC is linked to an automatically generated UUID (Universally Unique Identifier) in the iTD app. This UUID serves as a pseudonymized identifier for the user, which is used throughout the backend architecture of the iTD-App, particularly on the MONDY platform. The UUID is automatically generated to ensure high security and global uniqueness while maintaining the anonymity of user data. All personal data are subject to confidentiality and can only be viewed by authorized persons.

Data collected from the iTD-App is stored in two databases, one for the pseudonymized demographic data and the other one for health data on the MONDY platform. The server systems are hosted in Germany. All data streams from the iTD-App are stored in a database, an IBM firestore©; the Fast Healthcare Interoperability Resources (FHIR®) spec 4.3.0 is used as the data schema. To access the database via a FHIR API (Application Programming Interface), authentication with a personal account (username and password) is required. Only project consortium members have access.

In a Machine Learning Operations (MLOps) system [48], the collected data is used to train statistical models. To do so, the raw measurement data from the FHIR system are synchronized with the Scientific Cluster infrastructure of Leipzig University. Therefore, deleted data (e.g., upon user request) will also be deleted on this system. The training of the statistical models is done in multiple consecutive steps (e.g., data cleaning, data pre-processing, missing data handling). All intermediate results (i.e., transformed data) are deleted immediately when the processing step has been finished. Finally, the trained model is stored in a database, including meta-information about which kind of data from which participant has been used to train this model. This ensures that models can be deleted if a participant requests their data to be deleted. We are examining for which scenarios differential privacy methods can be used sensibly to prevent information leakage from our models.

### 1.6 Data analysis

The current study does not encompass confirmatory hypothesis testing and has explorative character, aiming to provide proof-of-concept for the value of personalized monitoring and forecasting of depression severity in individual patients. Missing data will be handled with model-specific imputation when needed.

To answer RQI, i.e. “Can personal MS data be an objective marker of the a) daily and b) weekly self-reported depressive symptom severity?“, we will conduct multilevel correlations between depressive symptom severity (PHQ-2, PHQ-9) and features derived from MS data through the application of data-driven methods. Multilevel approaches can be applied for statistical analyses of idiographic data [49,50]. Multilevel correlations will include participants as fixed effects and as random effect. All MS features will be included in the mixed model as fixed effects. However, in multilevel models, within-person variability is pooled across individuals, rather than person-specific [51]. Therefore, we will repeat analysis for each participant separately (truly idiographic *N* = 1 version of the analysis) to understand whether there are any differences in results if the data base is limited to MS features derived from personal data of the individual only. Apart from the correlations themselves, the effect sizes will be assessed to understand the clinical relevance of associations and a Random Forest classifier will be used to account for non-linear associations and to asses feature importance using Kernel SHAP analysis [52]. To validate our findings on an idiographic level, we will explore the use of unified structural equation modelling (USEM, [53,54]), including Group Iterative Multiple Model Estimation (GIMME; [55,56]) that can estimate both group-level relationships and individual-specific relationships.

To address RQII, i.e. “Can personal MS data predict the self-reported depressive symptom severity on a) the next day(s) and/or b) the next week(s)?“, we apply various machine learning techniques. Exploratory analyses investigate the predictability of depressive symptoms within individuals, using either classification or regression approaches. We compare idiographic models, which use data from individual patients, with nomothetic models based on data from all participants. Furthermore, we plan to examine whether multitask-learning methods [57] can enhance performance. Modern machine learning models, such as deep neural networks (DNNs), are validated against classical state-of-the-art methods, including boosted ensemble techniques [58], support vector machines [59], and autoregressive models [32] focusing on vector autoregressive (VAR) models and their analyses using forecast error variance decomposition (FEVD) and impulse response functions (IRF) specifically for idiographic approaches. Individual thresholds for high depressive symptom severity are derived from the data. Using eXplainable AI (XAI) methods, we evaluate the sensor technology component within iTD to understand the significance and impact of various MS features on the model's outcomes. This will help to identify the most relevant aspects of the MS data and clarify their influence on potential decision-making processes. Models based on data from all participants are validated using leave-one-subject-out cross-validation (LOSO-CV) [60,61], while within-subject models employ time-series cross-validation [62,63] and rolling back cross-validation [64].

For both RQ, sensitivity analyses will be conducted using different amounts of input data (e.g., 12 vs. 24 h of MS data, all AA vs. only MS data).

## 2 Discussion

This study aims to deepen the understanding of markers and predictors of individual disease trajectories in patients with depressive disorders. If changes in depressive symptoms can be predicted ahead of time, individuals could try to counteract depression relapse through timely self-management or treatment. They can gather MS data with little intrusion in their daily lives and based on these, self-management guidance and professional interventions for depression shall be offered in a tailored way and at the precise time when they are needed [65].

There are still some challenges to overcome to deploy MS data for facilitating timely self-management advice. One key challenge is deriving meaningful information from the huge datasets generated by MS. Data-driven methods are increasingly used to analyze complex associations, focusing on nonlinear models that identify patterns and dependencies in the input data. DNNs are capable of automatically learning abstractions in the data that can be entirely novel and have demonstrated superior performance over classical machine learning models across a range of tasks [66]. One notable limitation of DNNs, however, is their reliance on large and diverse datasets to achieve optimal performance. Yet, our study includes data from fewer than 30 patients, which may result in bad generalization [67]. Nonetheless, DNNs can still be used to generate complex features (e.g., activity metrics from raw accelerometer data) where sufficient data is available [68]. Additionally, pretrained models can be fine-tuned using transfer learning to mitigate the limitations of small datasets [69]. Another key concern of DNNs is their reputation as a “black box” approach — i.e., they are said to lack transparency or interpretability of how input data are transformed to model outputs [66]. Interpretability is crucial though in fields in which these algorithms' predictions have serious implications, such as psychiatry and digital mental health applications [70]. Where data relationships are inherently complex and probabilistic, enhancing the understandability of AI models through transparency and interpretability is essential to develop trustworthy systems fit for deployment [71]. XAI allows users and parts of the internal system to be more transparent, providing explanations of their decisions in some level of detail. These explanations are important to ensure algorithmic fairness, identify potential bias/problems in the training data, and to ensure that the algorithms perform as expected. Advancing research on XAI is crucial to successfully use MS data for patients’ daily self-management and treatment.

Regulatory and legal aspects represent yet another challenge for the successful deployment of MS data by patients with depression. The international ISO standard (ISO/TS 82304–2:2021), the GDPR and other EU regulations, such as the MDR (Medical Device Regulation) impact the development of mental health applications in European countries [72]. Academic research and product development are today more intertwined than ever, placing the research of fundamental questions into the framework of early product development of health apps [73]. Ensuring high patient protection significantly increases the costs required for clinical research, which raises the question of additional funding for such applications, especially to meet Europe's high regulatory standards.

Participants in the current, purely observational study are going to benefit from their study participation through the continuous self-monitoring of depressive symptoms. This provides a long-term documentation of changes in depressive symptoms, increasing self-awareness and providing new insights towards the self-management of their condition. Self-monitoring and journaling are tools that have been used traditionally for the treatment of any behavioural or emotional problem. Whilst the act of tracking *physical* symptoms resulted in greater symptom reporting in healthy subjects [74], previous studies reported beneficial effects of symptom tracking for *mental* health, including increased self- and symptom awareness [75,76], which in turn facilitated self-reflection and more proactive coping with symptoms [76,77]. Importantly, asking about suicidal thoughts has been shown to *not* provoke suicidal ideation [78].

### 2.1 Strengths and limitations

A major strength of the proposed study protocol is the focus on intraindividual processes and the personalization of the data analysis approach. However, there are also some limitations to address: (1) We hope to achieve a very high data density for the individual participants through continuous data collection over the course of one year and constant monitoring of data completeness and quality by the study centre. Potential missing data might pose limitations on the success of the study though. We require daily and weekly self-reports from patients as an indicator of their depressive symptom severity, which bears a risk of non-compliance or fatigue with the study procedures. If patients were more non-compliant on days with stronger depressive symptoms, the prediction of these days with higher symptom burden might get very difficult as this represented an “out-of-sample” prediction in Machine Learning terms. (2) The performance of prediction models may vary across participants and the focus on the individual furthermore bears a risk of overfitting predictive models. Even when we collect contextual information from participants, the data might not fully represent the daily experiences and context of the person, which makes it possible that patterns are related to the specific context of data collection during the year and might not generalize to the life of individuals after participation in the study. (3) The current work is a pilot study for individualized long-term depression modelling. Given the heterogeneity of depression, the symptoms and patterns observed in the current, small sample may not be representative of the broader population of patients suffering from depressive disorders. This limits the generalizability of our findings, which will need to be validated in larger samples.

### 2.2 Implications and future directions

To conclude, our study pursues a novel approach to enable patients in the future to use their existing personal data from smartphones and wearables for their disease management. The MONDY project consortium is committed to working towards high interoperability of the developed system, so that it could potentially be integrated into regular care (e.g. as electronic patient records) without major time lags from research to practice [79]. Using their own personal data for a better understanding of their depressive disorder could be empowering for the many individuals living with depression.

## CRediT authorship contribution statement

**Hanna Reich:** Writing – review & editing, Writing – original draft, Project administration, Methodology, Funding acquisition, Formal analysis, Conceptualization. **Simon Schreynemackers:** Writing – review & editing, Visualization, Project administration, Methodology, Formal analysis, Data curation. **Rebeka Amin:** Writing – review & editing, Methodology, Investigation. **Sascha Ludwig:** Writing – review & editing, Software, Project administration, Formal analysis, Data curation. **Jil Zippelius:** Writing – review & editing, Investigation. **Johannes Leimhofer:** Writing – review & editing, Formal analysis, Data curation. **Tobias Dunker:** Writing – review & editing, Software, Project administration, Data curation. **Elisabeth Schriewer:** Writing – review & editing, Project administration, Methodology. **Angela Carell:** Writing – review & editing, Supervision, Funding acquisition. **Yvonne Weber:** Writing – review & editing, Supervision, Methodology, Funding acquisition. **Ulrich Hegerl:** Writing – review & editing, Supervision, Methodology, Funding acquisition, Conceptualization.

## Funding

This work was supported by the German Federal Ministry of Education and Research (BMBF, grant numbers: 13GW0576D, 13GW0576A, 13GW0576B, 13GW0576C, date awarded: November 01, 2021).

## Declaration of competing interest

The authors declare that they have no known competing financial interests or personal relationships that could have appeared to influence the work reported in this paper.

## Data Availability

No data was used for the research described in the article.
