# Supplementary material for: Links between self-monitoring data collected through smartphones and smartwatches and the individual disease trajectories of adult patients with depressive disorders: Study protocol of a one-year observational trial
Source: Contemp Clin Trials Commun. 2025 May 10;45:101492. doi: 10.1016/j.conctc.2025.101492 (PMC12148402; doi:10.1016/j.conctc.2025.101492)
Supplement: Multimedia component 1 [file mmc1.pdf]

# Links between self-monitoring data collected through smartphones and smartwatches and the individual disease trajectories of adult patients with depressive disorders: Study protocol of a one-year observational trial

## Supplementary Material

Here, we report wordings of all items that were used for (1) the daily self-report and (2) as stimulus material for audio recordings. The original, German, version is followed by a simple translation to English language (provided in brackets).

### (1) Daily self-report

Twice a day, participants are asked to enter some information via self-report in the iTD app. The morning log is available from 6 a.m. to 12 p.m., and the evening log is available from 6 p.m. to midnight. Participants can set a reminder in the app to complete the logs. The following table provides an overview of the questionnaires and the time of assessment.

| | Questionnaire | Time of assessment |
|---|---|---|
| **a.** | Erleben / Fühlen / Befinden **(**Experience / Feeling / Well-being) | Morning and evening |
| **b.** | Schlafreport (Sleep report) | Morning |
| **c.** | Patient Health Questionnaire-2 | Evening |
| **d.** | Substanzkonsum (Substance use) | Evening |
| **e.** | Medikamenteneinnahme (Taking medication) | Evening |
| **f.** | Tagesaktivitäten (Daily activities) | Evening |

#### *a. Erleben / Fühlen / Befinden (Experience / Feeling / Well-being)*

*W*ie geht es Ihnen heute Morgen/Abend in Bezug auf… (How are you doing this morning/evening in relation to…)

| Item | Answering options (Visual Analogue Scale) |
|---|---|
| Ihre Anspannung? (Your tension?) | 0 = entspannt, 10 = angespannt (0 = relaxed, 10 = tense) |
| Ihre Erschöpfung? (Your exhaustion?) | 0 = erholt, 10 = erschöpft (0 = recovered, 10 = exhausted) |
| Ihre Gedanken? (Your thoughts?) | 0 = klar, konzentriert, 10 = kreisende Gedanken, starkes Grübeln (0 = clear, concentrated, 10 = circular thoughts, intense brooding) |
| Ihre Zuversicht? (Your confidence?) | 0 = pessimistisch, 10 = zuversichtlich, optimistisch (0 = pessimistic, 10 = confident, optimistic) |
| Gefühle von Traurigkeit? (Feelings of sadness?) | 0 = nicht vorhanden, 5 = bedrückt, 10 = tief traurig (0 = not present, 5 = depressed, 10 = deeply sad) |
| Ein Gefühl von Ärger? (A feeling of anger?) | 0 = nicht vorhanden, 5 = genervt, 10 = wütend (0 = not present, 5 = annoyed, 10 = angry) |
| Angstgefühle? (Feelings of anxiety?) | 0 = nicht vorhanden, 5 = besorgt, 10 = Panik (0 = not present, 5 = worried, 10 = panic) |
| Empfinden von Freude? (Feeling of joy?) | 0 = nicht vorhanden, 5 = zufrieden, 10 = glücklich (0 = not present, 5 = satisfied, 10 = happy) |

#### *b. Schlafreport (Sleep report)*

Wie viel Zeit haben Sie in der letzten Nacht verbracht mit… (How much time did you spend last night with…)

| Item | Answering options |
|---|---|
| Zeit bis zum Einschlafen (Time to fall asleep) | Freie Angabe in 15 Minuten-Intervallen (Free entry in 15-minute intervals) |
| Schlafen (Sleep) | Freie Angabe in 15 Minuten-Intervallen (Free entry in 15-minute intervals) |
| Nach dem Einschlafen wieder wach sein (Be awake again after falling asleep) | Freie Angabe in 15 Minuten-Intervallen (Free entry in 15-minute intervals) |

### *c. Patient Health Questionnaire-2*

(see description and references in the manuscript)

### *d. Substanzkonsum (Substance use)*

| Item | Answering options | | | |
|---|---|---|---|---|
| Haben Sie heute Koffein konsumiert? (Have you consumed caffeine today?) | Ja (Yes) | Nein (No) | | |
| Wenn ja, wie viele koffeinhaltige Getränke haben Sie getrunken? Mit koffeinhaltigen Getränken ist eine Tasse Kaffee, eine Tasse grüner oder schwarzer Tee, ein Glas Cola oder eine kleine Dose Energy Drink gemeint. (If so, how many caffeinated beverages did you consume? Caffeinated beverages include a cup of coffee, a cup of green or black tea, a glass of cola, or a small can of energy drink.) | 1 Getränk (1 drink) | 2-3 Getränke (2-3 drinks) | 4 oder mehr Getränke (4 or more drinks) | |
| Haben Sie heute Alkohol getrunken? (Have you drunk alcohol today?) | Ja (Yes) | Nein (No) | | |
| Wenn ja, wie viele alkoholische Getränke haben Sie schätzungsweise getrunken? Mit einem alkoholischen Getränk ist eine kleine Flasche Bier (0,33 l), ein kleines Glas Wein oder Sekt (0,125 l), ein doppelter Schnaps oder Likör (4 cl) oder ein Mixgetränk (0,33 l) gemeint. (If so, how many alcoholic drinks did you estimate you consumed? An alcoholic drink is defined as a small bottle of beer (0.33 l), a small glass of wine or sparkling wine (0.125 l), a double shot of schnapps or liqueur (4 cl), or a mixed drink (0.33 l).) | 1 Getränk (1 drink) | 2-3 Getränke (2-3 drinks) | 4-5 Getränke (4-5 drinks) | 6 oder mehr Getränke (6 or more drinks) |
| Haben Sie heute Tabak konsumiert? (Have you consumed tobacco today?) | Ja (Yes) | Nein (No) | | |
| Wenn ja, wie oft haben Sie geraucht? Gemeint sind neben Zigaretten auch Zigarillos, Zigarren, Pfeifen, Vapes, E-Zigaretten etc., die Tabak enthalten. (If so, how often did you smoke? This includes cigarettes, cigarillos, cigars, pipes, vapes, e-cigarettes, etc., which contain tobacco.) | Freie Angabe (Free entry) | | | |
| Haben Sie heute andere Substanzen oder Drogen konsumiert? (Have you consumed any other substances or drugs today?) | Ja (Yes) | Nein (No) | | |
| Wenn ja, welche? (If so, which ones?) | Freie Angabe (Free entry) | | | |

### *e. Medikamenteneinnahme (Taking medication)*

At the beginning of use of the study, the medications being taken, including the prescription schedule, are recorded in the individual profile in the iTD-App. This information can be modified by participants over the course of the study. The daily self-report items for medication intake are:

| Item | Answering options | |
|---|---|---|
| Haben Sie heute Ihre Medikamente planmäßig eingenommen? (Did you take your medication as scheduled today?) | Ja (Yes) | Nein (No) |
| (Falls Nein:) Tragen Sie bitte die Abweichung in die Textnotiz ein. Wurde Ihr Medikationsplan geändert? Dann geben Sie bitte die neue Medikation in ihrem Profil an. (If no:) Please enter the deviation in the text note. Has your medication plan been changed? Then please enter the new medication in your profile. | Freie Angabe (Free entry) | |

### *f. Tagesaktivitäten (Daily activities)*

Wie viel Zeit haben Sie heute mit den folgenden Aktivitäten verbracht? (How much time did you spend on the following activities today?)

| Item | Answering options |
|---|---|
| Arbeiten/ Studieren (Work/ study) | Freie Angabe in 15 Minuten-Intervallen (Free entry in 15-minute intervals) |
| Sport/ Bewegung (Sports/ exercise) | |
| Soziale Kontakte (Social contacts) | |
| Ausruhen/ Schlafen (Resting/ sleeping) | |
| Hausarbeit (Housework) | |
| Pflege anderer Menschen („Care-Arbeit“) (Caring for other people (“care work”) | |
| Freizeit/ Hobbies (Leisure/ hobbies) | |
| Zeit im Grünen/ draußen (Time outdoors/ in the countryside) | |

## (2) Stimulus material for voice recordings

| |
|---|
| Welche Art von kreativer Ausdrucksform, sei es Malen, Schreiben oder Musik, hat heute Ihren Tag bereichert? Was war der Inhalt der Ausdrucksform? (What kind of creative expression, be it painting, writing, or music, enriched your day today? What was the content of the expression?) |
| Suchen Sie einen besonderen Gegenstand in Ihrer Umgebung und beschreiben Sie ihn möglichst genau. (Find a special object in your surroundings and describe it as accurately as possible.) |
| Wie möchten Sie den Rest des Tages verbringen? Gibt es etwas, das Sie besonders gerne tun würden? (How would you like to spend the rest of the day? Is there anything you'd particularly like to do?) |
| Was haben Sie gestern zum ersten Mal gemacht? Beschreiben Sie es. Es kann auch nur eine Kleinigkeit sein. (What did you do for the first time yesterday? Describe it. It can be just a small thing.) |
| Welche Kleidung tragen Sie im Moment? Beschreiben Sie bitte Ihr Outfit von heute. (What are you wearing right now? Please describe your outfit today.) |
| Wie haben Sie in der letzten Nacht geschlafen? Gab es etwas Besonderes, das Sie ausgeruht oder vielleicht unruhig gemacht hat? (How did you sleep last night? Was there anything in particular that left you rested or perhaps uneasy?) |
| Was sind Ihre Pläne für morgen? Es muss nichts Besonderes sein. Schildern Sie sie. (What are your plans for tomorrow? It doesn't have to be anything special. Describe them.) |
| Welche Nachricht oder Information hat Sie in den letzten 24 Stunden überrascht? (What news or information surprised you in the last 24 hours?) |
| Welches Buch, welche Zeitschrift oder welchen Artikel haben Sie heute gelesen oder angefangen zu lesen? (What book, magazine or article did you read or start reading today?) |
| Wie gefällt Ihnen das Wetter heute? Beschreiben Sie es so ausführlich wie möglich. (How do you like the weather today? Describe it in as much detail as possible.) |
| Welche Aktivität hat heute Ihre Kreativität oder Neugierde angeregt? (What activity stimulated your creativity or curiosity today?) |
| Was waren heute drei gute Dinge des Tages und warum? (What were three good things about the day and why?) |
| Was war gestern ein bemerkenswertes Ereignis? Beschreiben Sie es, auch wenn es aus Ihrer Sicht eine Kleinigkeit ist. (What was a notable event yesterday? Describe it, even if it seems minor to you.) |
| Wie sind Sie heute in den Tag gestartet? (How did you start your day today?) |
| Was haben Sie gestern getan, um sich sicher und geborgen zu fühlen? Wenn Sie sich an nichts konkretes erinnern - was nehmen Sie sich für heute vor? (What did you do yesterday to feel safe and secure? If you don't remember anything specific, what are your plans for today?) |
| Welchen Song oder welche Musik haben Sie heute gehört? Um was geht es in dem Song? (What song or music did you listen to today? What is the song about?) |
